# mPGES-1 null mice are resistant to bleomycin-induced skin fibrosis

**DOI:** 10.1186/ar3226

**Published:** 2011-01-25

**Authors:** Matthew R McCann, Roxana Monemdjou, Parisa Ghassemi-Kakroodi, Hassan Fahmi, Gemma Perez, Shangxi Liu, Xu Shi-wen, Sunil K Parapuram, Fumiaki Kojima, Christopher P Denton, David J Abraham, Johanne Martel-Pelletier, Leslie J Crofford, Andrew Leask, Mohit Kapoor

**Affiliations:** 1 The Canadian Institute of Health Research Group in Skeletal Development and Remodeling, Division of Oral Biology and Department of Physiology and Pharmacology, Schulich School of Medicine and Dentistry, University of Western Ontario, Dental Sciences Building, London, Ontario, N6A 5C1, Canada; 2 Osteoarthritis Research Unit, University of Montreal Hospital Research Center (CR-CHUM) and Department of Medicine, University of Montreal, 1560 Rue Sherbrooke Est, Montréal, Québec, H2L 4M1, Canada; 3 Centre for Rheumatology, Department of Medicine, University College London (Royal Free Campus), Rowland Hill Street, London, NW3 2PF, UK; 4 Division of Rheumatology, Department of Internal Medicine, University of Kentucky, 740 S. Limestone Street, J-509 Kentucky Clinic, Lexington, KY 40536, USA

## Abstract

**Introduction:**

Microsomal prostaglandin E2 synthase-1 (mPGES-1) is an inducible enzyme that acts downstream of cyclooxygenase (COX) to specifically catalyze the conversion of prostaglandin (PG) H_2 _to PGE_2_. mPGES-1 plays a key role in inflammation, pain and arthritis; however, the role of mPGES-1 in fibrogenesis is largely unknown. Herein, we examine the role of mPGES-1 in a mouse model of skin scleroderma using mice deficient in mPGES-1.

**Methods:**

Wild type (WT) and mPGES-1 null mice were subjected to the bleomycin model of cutaneous skin scleroderma. mPGES-1 expressions in scleroderma fibroblasts and in fibroblasts derived from bleomycin-exposed mice were assessed by Western blot analysis. Degree of fibrosis, dermal thickness, inflammation, collagen content and the number of α-smooth muscle actin (α-SMA)-positive cells were determined by histological analyses. The quantity of the collagen-specific amino acid hydroxyproline was also measured.

**Results:**

Compared to normal skin fibroblasts, mPGES-1 protein expression was elevated in systemic sclerosis (SSc) fibroblasts and in bleomycin-exposed mice. Compared to WT mice, mPGES-1-null mice were resistant to bleomycin-induced inflammation, cutaneous thickening, collagen production and myofibroblast formation.

**Conclusions:**

mPGES-1 expression is required for bleomycin-induced skin fibrogenesis. Inhibition of mPGES-1 may be a viable method to alleviate the development of cutaneous sclerosis and is a potential therapeutic target to control the onset of fibrogenesis.

## Introduction

Scleroderma (systemic sclerosis, or SSc) is a fibrotic diseases for which there is currently no approved treatment [1]. Although the underlying causes are unknown, fibrotic disease is associated with the production and accumulation of excessive fibrous connective tissue and can be considered to arise because of an inability to appropriately terminate the normal wound repair response [2,3]. SSc is a prototypic multisystem and multistage fibrotic disease and is considered to be initiated by a combination of microvascular injury, inflammation, and autoimmunity, culminating in fibroblast activation and fibrosis [3]. Histological analysis of the initial stage of scleroderma reveals perivascular infiltrates of mononuclear cells in the dermis, and these infiltrates are associated with increased collagen synthesis in the surrounding fibroblasts [4,5]. Thus, understanding how to control the inflammatory stage of SSc may be of benefit in controlling the progression of early-onset disease.

Microsomal prostaglandin E_2 _synthases (mPGESs) are enzymes that catalyze the conversion of PGH_2 _to PGE_2 _[6]. Thus far, three PGE synthases - namely cytosolic PGE synthase (cPGES), mPGES-1, and mPGES-2 - have been characterized [6-8]. cPGES is localized in the cytosolic region of cells and tissues under basal conditions and is most likely to be involved in the homeostatic production of PGE_2 _[8]. mPGES-2 is also constitutively expressed in a wide variety of tissues and cell types and is synthesized as a Golgi membrane-associated protein [9]. In contrast, mPGES-1 is induced in response to inflammation and acts downstream of cyclooxygenases [10,11].

mPGES-1 has been shown to be a critical mediator of inflammation, pain, angiogenesis, fever, bone metabolism, and tumorgenesis [12-15]. We have previously shown that mPGES-1 expression is elevated in tissues and cells of various inflammatory diseases, including rheumatoid arthritis and osteoarthritis [10,11,16,17]. mPGES-1 null mice are resistant to chronic inflammation of joints in the models of collagen-induced arthritis (CIA) and collagen antibody-induced arthritis [12,13]. We recently showed that mPGES-1 is induced during the skin wound healing process in mice [18]. However, the expression and role of mPGES-1 in fibrogenesis are unknown.

There is no perfect mouse model that recapitulates every facet of SSc; however, the bleomycin-induced model of skin scleroderma is often used. In this model, repeated application of bleomycin, an anti-tumor antibiotic originally isolated from the fungus *Streptomyces verticillus *[19], is used to induce inflammation and subsequent fibrosis in skin [20]. Thus, the bleomycin model of skin SSc can be used to evaluate the potential role of individual genes in the early onset (or inflammatory phase) of SSc. The aim of the present study was first to examine whether mPGES-1 shows altered expression in fibroblasts isolated either from dermal lesions of patients with SSc or from mouse skin response to bleomycin and then to assess the potential role of mPGES-1 in the early phases of SSc by subjecting mice deficient in mPGES-1 to the bleomycin model of skin scleroderma [21].

## Materials and methods

### mPGES-1 null mice

mPGES-1 heterozygous (Het) male and female mice on a DBA1 lac/J background were provided by Pfizer Inc (Groton, CT, USA) [13]. mPGES-1 Het mice were mated to generate mPGES-1 null, Het, and littermate wild-type (WT) mice. All of the experiments were performed under the guidelines of the Institutional Animal Care and Use Committee. Genotypes were identified by polymerase chain reaction (PCR) of tail biopsy DNA extract by using two-primer sets for the mPGES-1 null allele (PGES-N257R, 5'-TGCTACTTCCATTTGTCACGTC-3' and PGES-4407R, 5'-TCCAAGTACTGAGCCAGCTG-3') and the WT allele (PGES-WT-F, 5'-TCCCAGGTGTTGGGATTTAGAC-3' and PGES-WT-R, 5'-TAGGTGGCTGTACTGT TTGTTGC-3') (Invitrogen Corporation, Carlsbad, CA, USA). After initial denaturation at 95°C for 15 minutes, PCR involved 40 cycles of 30 seconds at 95°C, 30 seconds at 56°C, and 45 seconds at 72°C, followed by elongation for 5 minutes at 72°C. DNA from mPGES-1 WT mice showed one band (412 base pairs [bp]), DNA from mPGES-1 null mice showed one band (720 bp), and DNA from mPGES-1 Het mice showed bands of both 412 and 720 bp [22].

### Bleomycin treatment

Bleomycin treatment was performed as previously reported [23,24]. Briefly, bleomycin (Sigma-Aldrich, St. Louis, MO, USA), diluted to 0.1 U/mL with phosphate-buffered saline (PBS), was sterilized with filtration. One hundred microliters of bleomycin or PBS was injected subcutaneously into a single location on the shaved back of mPGES-1 WT and null mice once daily for 4 weeks. Mice were killed by CO_2 _euthanasia after 4 weeks, and skin samples were collected for histology, immunohistochemistry, hydroxyproline assay, and Western blotting.

### Histological assessment of collagen content

Sections (0.5 μm) were cut with a microtome (Leica Microsystems, Wetzlar, Germany) and collected on Superfrost Plus slides (Fisher Scientific, Pittsburgh, PA, USA). Sections were then de-waxed in xylene and rehydrated by successive immersion in descending concentrations of alcohol. To assess the effects of mPGES-1 genetic deletion on collagen synthesis, trichrome collagen stain was employed as previously described [23,24]. Briefly, collagen content in each section was assessed by three blinded observers who used the following assessment criteria: 0 signifies no collagen fibres, 1 signifies few collagen fibres, 2 signifies a moderate amount of collagen fibres, and 3 signifies an excessive amount of collagen fibres. In addition, Northern Eclipse (Empix Imaging, Inc., Mississauga, ON, Canada) software was used to determine the dermal thickness in each stained section to account for changes in dermal thickness in WT and mPGES-1 null mice with or without bleomycin injection.

### Assessment of inflammation

To assess inflammation, the presence of macrophages in skin sections was detected by immunofluorescence with MOMA-2 (monocyte + macrophage marker) antibody (Abcam, Cambridge, UK), a marker for macrophage. Immunofluorescence was performed as previously described [25], and the number of macrophages was then counted. In addition, sections were stained with hematoxylin and eosin (H&E) (Fisher Scientific, Ottawa, ON, Canada). H&E staining was performed in accordance with the recommendations of the manufacturer. The effects of mPGES-1 genetic deletion on inflammation were graded on a scale of 0 to 3 by three separate blinded observers: 0 signifies no inflammatory cells, 1 signifies few inflammatory cells, 2 signifies moderate inflammatory cells, and 3 signifies extensive inflammatory cells.

### Alpha-smooth muscle actin immunohistochemistry

Sections were cut and processed as described above. Immunolabeling of alpha-smooth muscle actin (α-SMA) was performed with the DakoCytomation LSAB+ System-HRP kit (DakoCytomation, Carpinteria, CA, USA). Immunohistochemical procedures were performed in accordance with the recommendations of the manufacturer. Briefly, endogenous peroxide was blocked by using 0.5% H_2_O_2 _in methanol for 5 minutes. Non-specific IgG binding was blocked by incubating sections with bovine serum albumin (0.1%) in PBS for 1 hour and then was incubated with primary antibody for α-SMA (1:1,000) in a humidified chamber and left overnight at 4°C. Next, sections were incubated with a biotinylated link for 30 minutes followed by incubation with streptavidin for 30 minutes. The chromogen diaminobenzidine tetrahydrochloride (DAB) was then added until sufficient color developed, and sections were counterstained with Harris's hematoxylin.

### Hydroxyproline assay

Hydroxyproline assay was performed as a marker of collagen content in bleomycin-treated/untreated skin with the method previously described [26]. Skin tissues were homogenized in saline and hydrolyzed with 2N NaOH for 30 minutes at 120°C, and then we determined hydroxyproline content by modifying the Neumann and Logan's reaction with Chloramine T and Ehrlich's reagent with a hydroxyproline standard curve measuring at 550 nm. Values were expressed as micrograms of hydroxyproline per milligrams of protein.

### Cell culture, immunofluorescence, and Western analysis

Dermal mouse fibroblasts were isolated from explants (4- to 6-week-old WT and mPGES-1 null mice) as described [27]. Also, dermal fibroblasts were isolated from an explant culture of 4-mm punch biopsies from the forearm of healthy individuals and those with early-onset (between 3 and 18 months after initial diagnosis) diffuse cutaneous scleroderma (6 each) in Dulbecco's modified Eagle's medium and 10% fetal bovine serum (Invitrogen Corporation) as previously described [28]. Donors were age-, site-, and sex-matched. No patients were on immunosuppressants. Experimental protocols were approved by the ethics committee of the Royal Free Hospital (UK), where all participants were recruited under informed written consent and human experimentation was conducted. Cells were subjected to indirect immunofluorescence analysis, as previously described [29], by using anti-mPGES-1 antibody (Cayman Chemical Company, Ann Arbor, MI, USA) followed by an appropriate secondary antibody (Jackson ImmunoResearch Laboratories Inc., West Grove, PA, USA) and were photographed with a Zeiss Axiphot camera (Empix Imaging, Inc.). Alternatively, cells were lysed in 2% SDS, and proteins were quantified (Pierce, Rockford, IL, USA) and subjected to Western blot analysis as previously described [30]. The following primary antibodies were used for Western blotting: anti-mPGES-1 (Cayman Chemical Company, Charlotte, NC, USA), anti-α-SMA (Sigma-Aldrich), and anti-β-actin (Sigma-Aldrich).

### Statistical analysis

Statistical analysis was performed with a two-tailed analysis-of-variance test in conjunction with a *post hoc *Mann-Whitney *U *test. Results are expressed as the mean ± standard error. A *P *value of less than 0.05 was considered statistically significant (denoted by an asterisk).

## Results

### mPGES-1 is overexpressed in human dermal SSc fibroblasts and in bleomycin-induced skin sclerosis in mice

**Figure 1:**
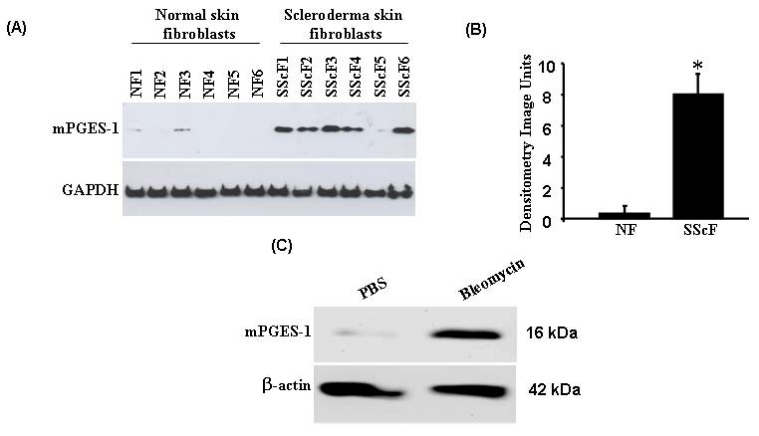
**mPGES-1 is induced in human systemic sclerosis (SSc) fibroblasts and in response to bleomycin-induced skin sclerosis**. **(a, b) **Western blot analysis showing upregulation in the expression of mPGES-1 in fibroblasts from the scars of SSc patients compared with fibroblasts from normal human skin (NF). Representative data from six separate cell lines per group are shown. **(c) **Western blot analysis showing induction of mPGES-1 in the skin of wild-type (WT) mice in response to 4-week treatment with bleomycin. Representative data from four separate animals per group are shown. mPGES-1, microsomal prostaglandin E_2 _sythnase-1.

To begin to assess whether mPGES-1 plays a role in fibrogenesis in SSc, we first examined whether mPGES-1 protein showed an altered expression pattern in dermal fibroblasts isolated from fibrotic lesions of early-onset diffuse SSc patients compared with those isolated from identical areas of healthy skin (termed normal fibroblast, or NF) (Figure 1a). Our results clearly showed that mPGES-1 protein was significantly upregulated in fibrotic fibroblasts from the skin of SSc patients compared with NFs isolated from healthy skin (Figure 1b). To continue our studies, we then evaluated whether mPGES-1 was induced *in vivo *in response to bleomycin-induced skin sclerosis. To do this, we injected WT mice subcutaneously for 4 weeks with bleomycin or PBS and skin biopsies were isolated 4 weeks post bleomycin or PBS treatment. From these, protein extracts were prepared and subjected to Western blotting with anti-mPGES-1 antibody (Figure 1c). Results showed that mPGES-1 was significantly induced in the skin in response to bleomycin as compared with PBS. Collectively, these results revealed that mPGES-1 is induced during fibrosis and may play a role in fibrogenesis.

### mPGES-1 genetic deletion results in reduced inflammation in response to bleomycin

**Figure 2:**
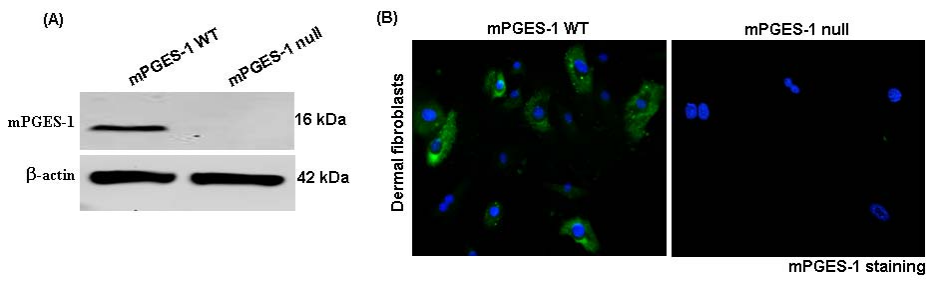
**Characterization of mPGES-1 genetic deletion**. **(a) **Western blot showing loss of mPGES-1 expression in dermal fibroblasts isolated from mPGES-1 null mice. Representative data from four separate cell lines per group are shown. **(b) **Dermal fibroblasts isolated from wild-type (WT) and mPGES-1 null mice were tested for the presence of the mPGES-1 by indirect immunofluorescence of cells with an anti-mPGES-1 antibody. Cells were counterstained with 4'-6-diamidino-2-phenylindole (DAPI) (blue) to detect nuclei. Representative data from four separate cell lines per group are shown. mPGES-1, microsomal prostaglandin E_2 _sythnase-1.

**Figure 3:**
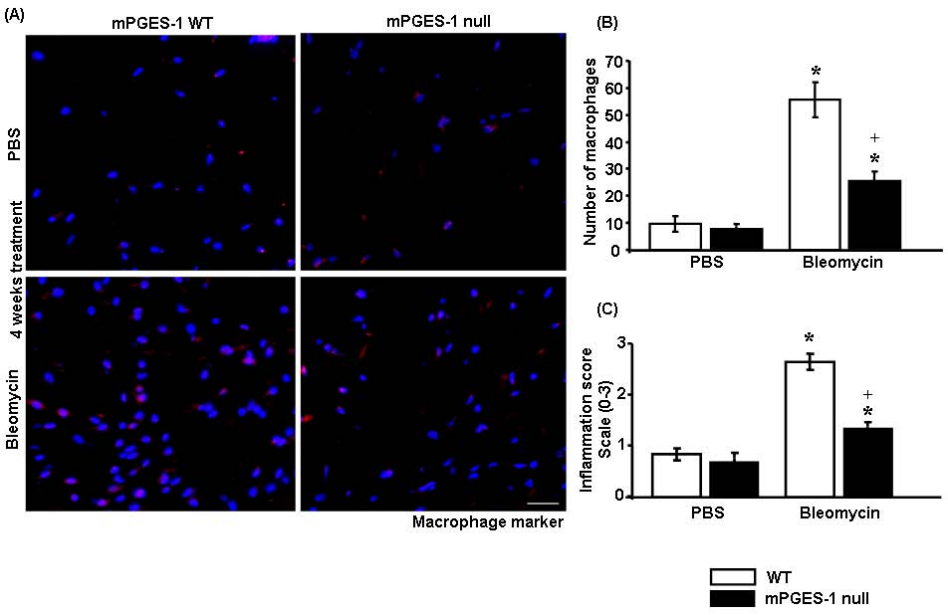
**mPGES-1 null mice exhibit reduced inflammation in **response **to bleomycin treatment**. **(a) **Immunofluorescence staining was performed with MOMA-2 antibody (a marker of macrophages) to account for inflammation in response to bleomycin treatment (4-week bleomycin treatment). **(b) **mPGES-1 null mice showed a marked reduction in the number of macrophages compared with the control mice in response to bleomycin. *P < 0.05; bleomycin-treated wild-type (WT) and mPGES-1 null mice compared with phosphate-buffered saline (PBS)-treated mice. ^+^*P *< 0.05, bleomycin-treated mPGES-1 null mice compared with bleomycin-treated WT mice. Representative data from four separate animals per group are shown. **(c) **Hematoxylin and eosin (H&E)-stained sections were further scored in a blinded fashion to account for inflammation as described in Materials and methods. mPGES-1 null mice showed a reduced inflammation score compared with WT mice in response to bleomycin. *P < 0.05; bleomycin-treated WT and mPGES-1 null mice compared with PBS-treated mice. ^+^*P *< 0.05; bleomycin-treated mPGES-1 null mice compared with bleomycin-treated WT mice. Representative data from four separate animals per group are shown. MOMA-2, monocyte + macrophage marker; mPGES-1, microsomal prostaglandin E_2 _sythnase-1.

After having demonstrated that mPGES-1 is overexpressed in fibrosis, we sought to assess whether mPGES-1 is required for fibrogenesis. Accordingly, we subjected WT and mPGES-1 null mice to the bleomycin model of skin scleroderma. Mice harboring a deletion of the mPGES-1 gene were detected by PCR analysis of tail DNA as previously described [22,30,31] and by subjecting dermal fibroblasts cultured from skin explants derived from WT and mPGES-1 null mice to Western blot and immunofluorescence analyses using an anti-mPGES-1 antibody (Figure 2a). Since mPGES-1 mediates inflammation *in vitro *as well as *in vivo *[22,30,31] and inflammation is involved with the onset of fibrogenesis [3,4,32], we employed indirect immunofluorescence analysis with an anti-MOMA-2 antibody (a marker of macrophages) to examine the effect of loss of mPGES-1 on the ability of bleomycin to induce the appearance of macrophages. As anticipated, we observed a marked increase in the number of macrophages in WT mice exposed to bleomycin compared with WT mice exposed to PBS (Figure 3a). However, compared with WT control mice, mPGES-1 null mice possessed markedly reduced numbers of macrophages in response to bleomycin (Figure 3a). Furthermore, semiquantitative blinded histological analysis of H&E-stained sections showed that bleomycin exposure resulted in a significantly lower inflammation score in mPGES-1 null mice compared with their WT counterparts (Figure 3c). Thus, loss of mPGES-1 resulted in a resistance to bleomycin-induced inflammation.

### Deletion of mPGES-1 results in resistance to bleomycin-induced collagen production and skin thickness

**Figure 4:**
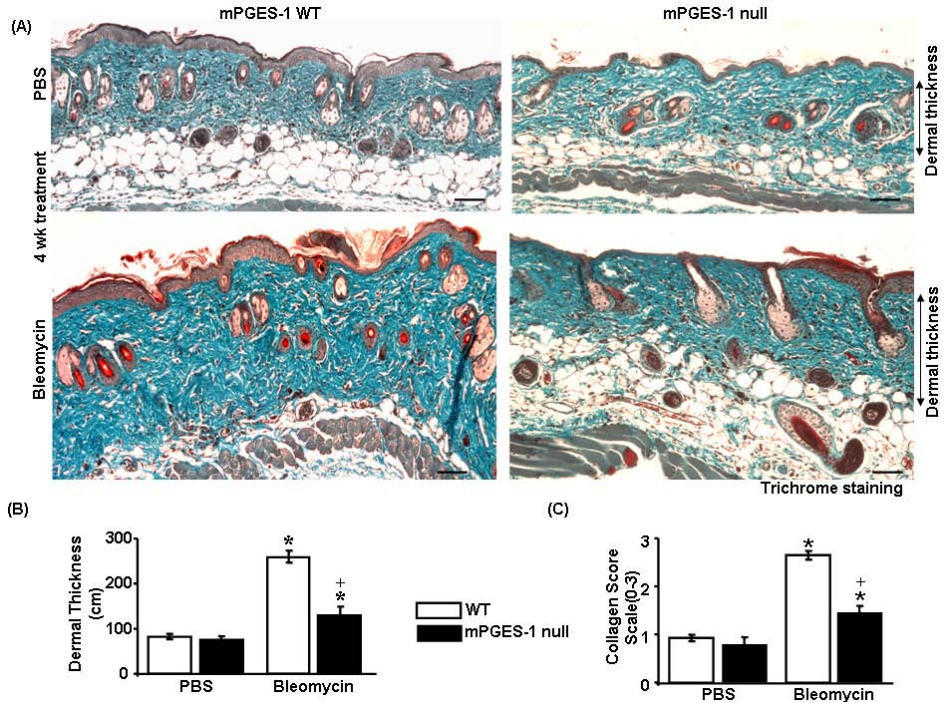
**mPGES-1 null mice show resistance to bleomycin-induced fibrosis *in **vivo*****. **(a) **Trichrome staining was performed to account for collagen content (degree of fibrosis) and dermal thickness in response to bleomycin treatment (4-week bleomycin treatment). **(b) **mPGES-1 null mice exhibited reduced dermal thickness compared with wild-type (WT) mice in response to bleomycin treatment. **(c) **Blind histological analysis in trichrome-stained sections showed that bleomycin-treated mPGES-1 null mice exhibited reduced collagen score compared with bleomycin-treated WT mice. *P < 0.05; bleomycin-treated WT and mPGES-1 null mice compared with phosphate-buffered saline (PBS)-treated mice. ^+^*P *< 0.05; bleomycin-treated mPGES-1 null mice compared with bleomycin-treated WT mice. Representative data from four separate animals per group are shown. mPGES-1, microsomal prostaglandin E_2 _sythnase-1.

**Figure 5:**
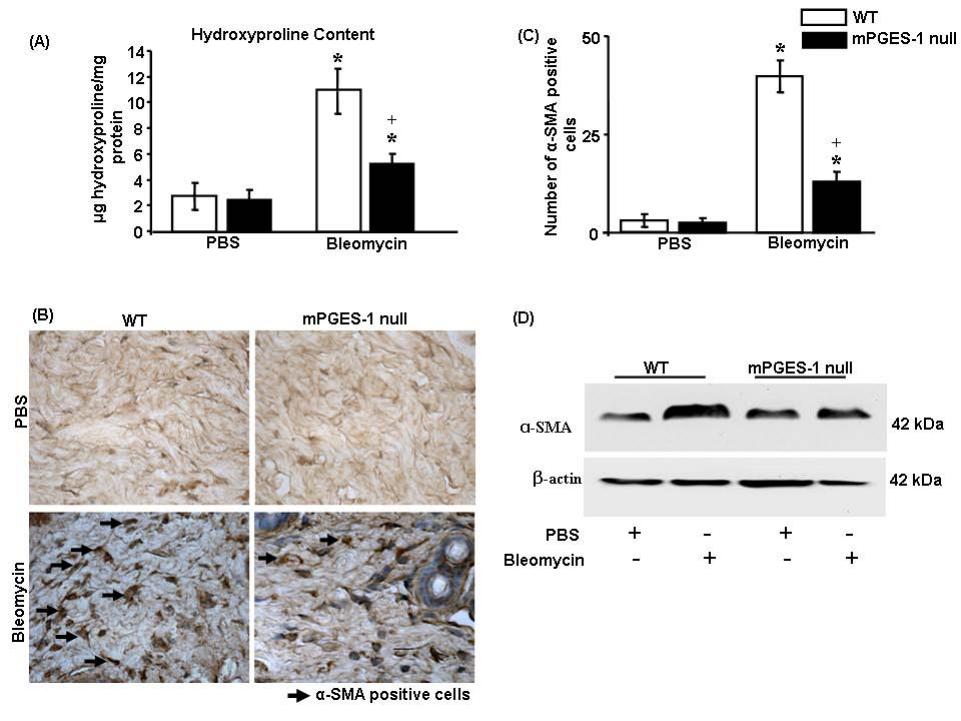
**mPGES-1 genetic deletion results in reduced collagen content and myofibroblast formation *in vivo***. **(a) **Hydroxyproline analysis showed reduced collagen content in mPGES-1 null mice compared with wild-type (WT) mice in response to bleomycin treatment. Data from four separate mice per group are shown. **(b, c) **Immunohistochemistry using anti-α-SMA antibody was performed. mPGES-1 null mice showed a reduced number of α-SMA-expressing myofibroblasts compared with WT mice in response to bleomycin treatment (4-week treatment). Representative data from four separate animals per group are shown. *P < 0.05; bleomycin-treated WT and mPGES-1 null mice compared with phosphate-buffered saline (PBS)-treated mice. ^+^*P *< 0.05; bleomycin-treated mPGES-1 null mice compared with bleomycin-treated WT mice. **(d) **Protein extracts from skin tissue after 4 weeks of bleomycin or PBS treatment were subjected to Western blot analysis with an anti-α-SMA antibody. mPGES-1 null mice treated with bleomycin showed reduced α-SMA expression compared with bleomycin-treated WT mice. Representative blot from four separate animals per group is shown. α-SMA, alpha-smooth muscle actin; mPGES-1, microsomal prostaglandin E_2 _sythnase-1.

To probe whether, in mPGES-1 null mice, reduced bleomycin-induced inflammation corresponded with reduced fibrosis, we then investigated whether loss of mPGES-1 resulted in a resistance to bleomycin-induced matrix deposition. To perform this analysis, we subjected bleomcyin-exposed skin of WT and mPGES-1 null mice to histological and biochemical analyses. As anticipated, as visualized by H&E and trichrome staining and hydroxyproline/praline analyses, bleomycin treatment in WT mice resulted in significant increases in extracellular matrix (ECM) deposition, dermal thickness, collagen score, and collagen content (Figure 4a-c and 5a). However, mPGES-1 null mice were relatively resistant to bleomycin-induced dermal thickness, ECM deposition, collagen score, and collagen content (Figure 4a-c and 5a). We did not observe any significant difference in ECM deposition between WT and mPGES-1 null mice in response to PBS. Thus, mirroring the effect observed on bleomycin-induced inflammation, loss of mPGES-1 resulted in a resistance to bleomycin-induced ECM deposition.

### mPGES-1 genetic deletion results in reduced α-SMA expression in response to bleomycin

As α-SMA-expressing myofibroblasts are a hallmark of both SSc and bleomycin-induced skin fibrosis [23,24], we then continued our studies by determining the effect of loss of mPGES-1 on the induction of α-SMA-expressing myofibroblasts in response to bleomycin injection. To begin to perform these analyses, we first subjected skin sections of bleomycin- or PBS-exposed WT or mPGES-1 null mice to immunohistochemical analysis with an anti-α-SMA antibody. Compared with skin of WT mice injected with PBS, skin of WT mice injected with bleomycin possessed markedly elevated numbers of myofibroblasts (Figure 5b, c), and this is consistent with previously published data [23,24]. Conversely, mPGES-1 null mice were relatively resistant to the ability of bleomycin to induce α-SMA-expressing myofibroblasts (Figure 5b, c). Confirming these data, Western blot analysis on protein samples derived from WT and mPGES-1 null mice treated with PBS or bleomycin showed that bleomycin resulted in elevated α-SMA protein production in WT mice but not in mPGES-1 null mice (Figure 5d). Collectively, our data are consistent with the notion that loss of mPGES-1 expression confers resistance to bleomycin-induced skin fibrosis and that mPGES-1 may play a key role in inflammation-induced fibrogenesis.

## Discussion

Since its discovery in 1999 [6], mPGES-1 has been a target of anti-inflammatory drug therapy. mPGES-1 is induced in human synovial tissue in osteoarthritis patients and in animal models of inflammation such as full-thickness incisional models of wound healing [18], CIA [22], lipopolysaccharide (LPS)-induced pyresis, and adjuvant-induced arthritis [33,34]. Moreover, in a variety of mesenchymal cell types (including fibroblasts), mPGES-1 is induced by proinflammatory stimuli, including LPS, interleukin-1-beta (IL-1β), and tumor necrosis factor-alpha (TNF-α) [6,10,11,17,30,31,35]. These results suggest that mPGES-1 plays a key role in driving inflammation. Although a role for inflammation in fibrogenesis is well established, the *in vivo *role for mPGES-1 in fibrosis has not been reported thus far.

A potent and selective inhibitor for mPGES-1 is not yet commercially available; however, mice with genetic deletion for mPGES-1 do exist, and these mice have been useful to define the *in vivo *role of mPGES-1. Our present study uses the bleomycin-induced model of skin fibrosis to assess whether mPGES-1 is essential for the onset of fibrosis. To provide a clinical context for our studies, we first showed that mPGES-1 protein expression was elevated in SSc skin fibroblasts. We then showed that mPGES-1 was induced in response to bleomycin in mouse skin fibroblasts *in vivo*.

It is largely believed that enhanced inflammatory response is necessary for fibrogenesis [32]. Accumulating evidence indicates a critical involvement of infiltrating macrophages and T cells in the pathogenesis of SSc. High numbers of infiltrating activated macrophages and T cells have been detected in skin of patients with SSc [36,37] and these cells are key producers of a variety of pro-fibrotic cytokines such as transforming growth factor-beta (TGF-β), CC-chemokine ligand 2, and IL-4 and IL-17 [38-40]. Therefore, we investigated the effect of mPGES-1 genetic deletion on inflammatory response by detecting macrophage infiltration in response to bleomycin treatment. mPGES-1 null mice showed marked reduction in the number of macrophages (inflammation) in response to bleomycin treatment, supporting our previous findings that mPGES-1 is a critical mediator of inflammation [22]. In future studies, it would be very interesting to determine the different subsets of infiltrating macrophages regulated by mPGES-1 during SSc disease. In addition, it should be investigated whether and how T cells are regulated by mPGES-1 during SSc. Since this is beyond the scope of the present study, future studies need to be directed toward understanding these concepts.

After determining the effect of mPGES-1 on inflammation, we further investigated the effect of mPGES-1 deletion on the degree of skin fibrosis. mPGES-1 null mice showed a resistance to bleomycin-induced skin fibrosis, as visualized by reduced dermal thickness and collagen production. The myofibroblast is the major cell type believed to be responsible for fibrogenesis, including in SSc [27,41,42]. Compared with WT mice, mPGES-1 null mice had fewer myofibroblasts in response to bleomycin injection. Our results collectively suggest that genetic deletion of mPGES-1 suppresses fibrogenesis *in vivo*.

Bleomycin-induced fibrosis is an inflammation-driven model and it is well established that PGE_2_, the product of mPGES-1, is one of the major proinflammatory mediators upregulated during inflammation. Given the known role of mPGES-1 in driving inflammatory responses, our results strongly suggest that mPGES-1 may play a key role in the initial, inflammatory stages of SSc. Our present study demonstrates that mice lacking mPGES-1 show resistance to bleomycin-induced fibrogenesis and is consistent with the notion that inflammation is involved with the onset of fibrosis, including SSc [32,43,44]. However, it is well established that inflammation plays a biphasic role in fibrosis; for example, the inflammatory protein TNF-α plays a biphasic role in fibrogenesis by promoting the initiation/inflammatory stage of fibrogenesis but suppressing the later, fibrotic stage of fibrosis [45-49]. As a specific illustration, TNF-α suppresses the ability of TGF-β to induce connective growth factor (CTGF/CCN2) in dermal fibroblasts [45]. In this regard, it is interesting to note that PGE_2 _(the only known product of mPGES-1) and iloprost (a synthetic version of prostacyclin, or PGI_2_) have been repeatedly shown to exhibit antifibrotic effects in experimental models of established fibrosis, including reducing CCN2 and collagen production in normal and fibrotic dermal fibroblasts, at least in part, acting through a cAMP-mediated suppression of ERK (extracellular signal-regulated kinase) activation [50-55]. Indeed, it has been hypothesized that prostacyclins limit the activation of fibroblasts following tissue injury but, in response to the original injury, may promote recruitment of inflammatory cells and lead to secondary activation of fibroblasts [56]. Moreover, given these concerns (and consistent with our data showing that SSc fibroblasts overexpress mPGES-1), it is interesting to note that prostanoid (including PGE_2_) production was greatly elevated in scleroderma cells compared with control cells and, given that excess added prostenoids reduced collagen and CCN2 overexpression in SSc fibroblasts, may act to limit further increases in collagen and CCN2 levels in these cells [50]. Given these considerations, it is likely that although mPGES-1 may contribute to the initiation of fibrogenesis through its ability to promote inflammation, mPGES-1 may actually act to control the overexpression of profibrotic genes in established lesions [56]. Investigation of the role of mPGES-1 in established fibrosis (for example, using the tight skin [Tsk] mouse [57]) is beyond the scope of the present study.

## Conclusions

Identification of new targets to counteract fibrosis is critical as currently no satisfactory antifibrotic treatment is available. Our new data strongly suggest that, likely based on its essential role in driving inflammation, mPGES-1 may be considered a novel target that might be useful in slowing the initial, rapidly progressing, inflammatory phase of SSc that is required for the subsequent development of fibrosis and therefore may be useful in a stage-specific modulation of the pathogenesis of SSc.

## Abbreviations

α-SMA: alpha-smooth muscle actin; bp: base pairs; CIA: collagen-induced arthritis; cPGES: cytosolic prostaglandin E synthase; ECM: extracellular matrix; H&E: hematoxylin and eosin; Het: heterozygous; IL: interleukin; LPS: lipopolysaccharide; MOMA-2: monocyte + macrophage marker; mPGES-1: microsomal prostaglandin E_2 _sythnase-1; NF: normal fibroblast; PBS: phosphate-buffered saline; PCR: polymerase chain reaction; PGE_2_: prostaglandin E_2_; SSc: systemic sclerosis; TGF-β; transforming growth factor-beta; TNF-α: tumor necrosis factor-alpha; WT: wild-type.

## Competing interests

The authors declare that they have no competing interests.

## Authors' contributions

MK and AL had full access to all of the data in the study, shared responsibility for the integrity of the data and the accuracy of the data analysis, and contributed to study conception and design and to analysis and interpretation of data. MRM and RM contributed to study conception and design and to acquisition, analysis, and interpretation of data. LJC contributed to study conception and design. PG-K, GP, SL, XS-w, SKP, and FK contributed to acquisition, analysis, and interpretation of data. HF, CPD, DJA and JMP contributed to analysis and interpretation of data. All authors were involved in drafting the article or revising it critically for important intellectual content and read and approved the final manuscript.
